# Recent advances on anti-angiogenesis receptor tyrosine kinase inhibitors in cancer therapy

**DOI:** 10.1186/s13045-019-0718-5

**Published:** 2019-03-12

**Authors:** Shuang Qin, Anping Li, Ming Yi, Shengnan Yu, Mingsheng Zhang, Kongming Wu

**Affiliations:** 1 Department of Oncology, Tongji Hospital of Tongji Medical College, Huazhong University of Science and Technology, 1095 Jiefang Avenue, Wuhan, 430030 Hubei China; 2 Department of Oncology, The First Affiliated Hospital of Zhengzhou University, Zhengzhou, 450052 Henan China

**Keywords:** Anti-angiogenic, Tyrosine kinase inhibitors, VEGF, Immunotherapy

## Abstract

Angiogenesis has always been the topic of major scientific interest in the field of malignant tumors. Nowadays, targeting angiogenesis has achieved success in various carcinomas by several mechanisms, including the use of anti-angiogenic small molecule receptor tyrosine kinase inhibitors (TKIs). The development of TKIs targeting pro-angiogenic receptors, mainly vascular endothelial growth factor receptor (VEGFR) family, have significantly improved the outcome of certain types of cancers, like renal cell carcinoma, hepatocellular carcinoma, and colorectal carcinoma. However, the general response rate is not very satisfactory. The particular toxicity profile and resistance to anti-angiogenic targeted agents are unavoidable, and no specific marker is available to screen responsive patients to TKIs for precision therapy. To date, about 11 anti-angiogenic TKIs with different binding capacities to angiogenic receptor tyrosine kinase have been approved for the treatment of patients with advanced cancers. This review presents all approved anti-angiogenic small molecule receptor TKIs so far with an emphasis on their indications and clinical efficacy. We also discuss the combination between TKIs and immune checkpoint blockade inhibitors based on the most recent exciting outcome in immunotherapy.

## Background

As described by Hanahan and Weinberg, tumor angiogenesis is regarded as one of the ten hallmarks of cancer [1]. Neovasculature supplies tumor with essential nutrients and oxygen and removes waste produce especially for those whose size is larger than 1–2 mm [2]. Based on the theory that tumor growth, progression, and metastasis depend on angiogenesis, targeting tumor blood vessels has been introduced as a logical approach to the treatment of various malignancies [3]. Subsequently, enormous innovative anti-angiogenic agents have been developed and tested in clinical trials.

In physiological circumstances, angiogenesis is under a relatively dynamic homeostasis, tightly controlled by pro-angiogenic and anti-angiogenic regulators. In cancer, however, the balance of pro- and anti-angiogenic are disturbed, leading to the switch to angiogenesis [4]. Tumor angiogenesis is an intricate mechanism regulated by multiple signaling pathways. Certain well-known pro-angiogenic factors include vascular endothelial growth factor (VEGF) [5], angiopoietin (ANGPT) [6], basic fibroblast growth factor (bFGF) [7], and platelet-derived growth factor (PDGF) [8], while endogenous anti-angiogenic factors encompass endostatin [9], angiostatin [10], and so on. In the past few decades, the efforts to develop anti-angiogenic treatment mainly focus on inhibiting VEGF/VEGFR signaling pathway such as anti-VEGF antibody bevacizumab [11] and anti-VEGFR2 antibody ramucirumab [12]. Anti-VEGF/VEGFR single-target drugs often lead to transient responses; however, tumor progression happens because other pathways, such as the PDGF/ PDGFR, FGF/FGFR, and ANGPT/Tie-2, provide potential escape mechanisms [13]. Anti-angiogenic agents inhibiting multiple signaling pathways seem more promising; therefore, multiple pan-target agents have been developed [13].

The binding of ligand to its receptor, for instance, VEGF and VEGFR, initiates the activity of tyrosine kinase domain of receptor and upregulates the downstream signal system. Those kinases are often upregulated in cancer and regarded as attractive therapeutic candidates. By April 2017, among the 35 protein kinase inhibitors (PKIs) approved, 31 are used in cancer therapy [14]. For example, sorafenib, sunitinib, and pazopanib are approved anti-angiogenic small molecule tyrosine kinase inhibitors (TKIs). Several novel anti-angiogenic TKIs with promising preclinic outcome are being studied in phase III clinical trials. Furthermore, new advance on combination of immunotherapy and TKIs attracts attention for superior efficacy.

## Receptor tyrosine kinases and anti-angiogenic small molecule receptor TKIs

**Fig. 1:**
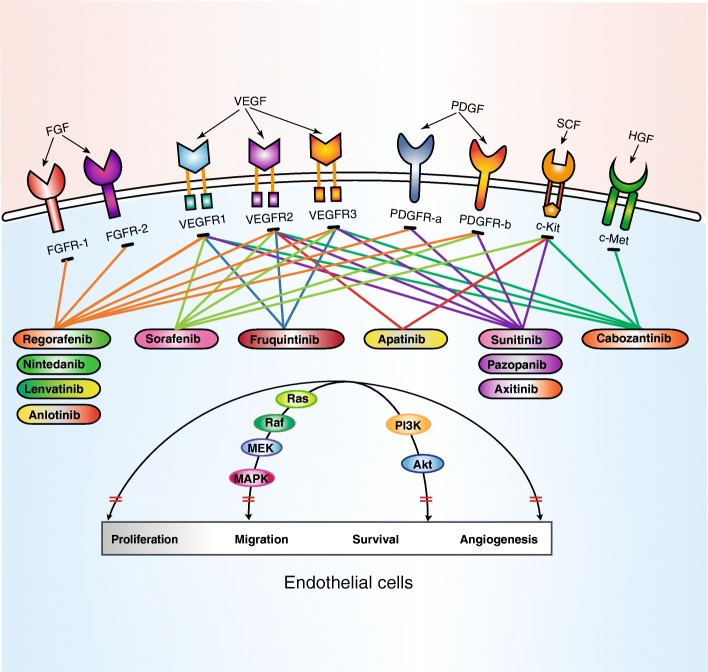
Main targets of approved anti-angiogenic receptor tyrosine kinase inhibitors (TKIs). All approved anti-angiogenic receptor TKIs can target multiple receptor sites simultaneously. The main targets included vascular endothelial growth factor receptor (VEGFR), platelet-derived growth factor receptor (PDGFR), fibroblast growth factor receptor (FGFR), c-Kit, and c-Met. Anti-angiogenic TKIs block the kinase activity of receptor and transduction of downstream signal involved in the proliferation, migration, and survival

There are 518 protein kinases encoded in the human genome, of which 90 kinases belong to the group of tyrosine kinases, being a major subclass of the human protein kinases [15, 16]. The tyrosine kinase group can be classified in receptor tyrosine kinases (RTKs) and non-receptor tyrosine kinases (nRTKs), and the former exerts function on the transduction of extracellular signals into the cell while the latter accomplishes intracellular communication [17]. RTKs share a great degree of similarity in their molecular structure, with an extracellular domain which can bind specific ligands, a single trans-membrane helix, and an intracellular region which contain a protein tyrosine kinase domain [18]. The intracellular domain, also called kinase domain, which is a bi-lobar structure, is composed of an N-terminal lobe, a C-terminal lobe, and an adenosine triphosphate (ATP) binding cleft located between them [19]. Ligand binding to the extracellular domain induces dimerization and allows auto-phosphorylation of the intracellular domains and activation of the receptor’s tyrosine kinase [20]. RTKs catalyze the transfer of the phosphates of ATP to the hydroxyl group of tyrosine residues on target proteins [21]. The RTK family includes the insulin receptor and the receptors for many growth factor families such as VEGF, FGF, PDGF, and epidermal growth factor (EGF) [21]. The VEGF-related gene family comprises six secreted proteins, namely VEGF-A, VEGF-B, VEGF-C, VEGF-D, VEGF-E, and placenta growth factor (PIGF) [22], and the VEGFR family consists of three related RTKs, VEGFR-1, VEGFR-2, and VEGFR-3 (Fig. 1) [23]. VEGF-A is the most important mediator, mediating its effects by binding to its two high-affinity RTKs: VEGFR-1 and VEGFR-2. PDGFs are the second important growth factor related to angiogenesis. There are at least four members in the PDGF family, namely PDGF-A, PDGF-B, PDGF-C, and PDGF-D. PDGFs act via two RTKs, known as PDGFR-α and PDGFR-β [24]. Meanwhile, bFGF, belonging to the FGF family, also contribute to angiogenesis [25]. The binding of ligand to its corresponding receptor initiates phosphorylation of the RTKs and leads to the activation of downstream signaling pathways, such as the PI3K/Akt and Ras/Raf/MEK/MAPK, which are involved in the proliferation, migration, and apoptosis of endothelial cells [26]. Therefore, small molecule inhibitors of RTKs are regarded as rational targets for cancer therapy.

## Approved anti-angiogenic receptor TKIs

Currently, angiogenic TKIs approved to cancer treatment by the US Food and Drug Administration (FDA) or China Food and Drug Administration (CFDA) are listed in Table 1.

**Table 1:** Principal clinical trials for the approval of anti-angiogenesis receptor tyrosine kinase inhibitors (TKIs)

Drug (company)	Indication	Pivotal study	Study design	PFS	OS	ORR	Approval time
Sorafenib (Bayer and Onyx)	RCC	NCT00073307 [27]	Phase III, sorafenib vs. placebo	HR = 0.44, *p* < 0.01	HR = 0.77, *p* = 0.02	10% vs. 2%	2005 (FDA)
	HCC	NCT00105443 [28]	Phase III, sorafenib vs. placebo	HR = 0.58, *p* < 0.001	HR = 0.69, *p* < 0.001	2% vs. 1%	2007 (FDA)
	DTC	NCT00984282 [29]	Phase III, sorafenib vs. placebo	HR = 0.59, *p* < 0.0001	HR = 0·80, *p* = 0·14	12.2% vs. 0.5%	2013 (FDA)
Sunitinib (Pfizer)	GIST	NCT00075218 [32]	Phase III, sunitinib vs. placebo	HR = 0.33, *p* < 0.0001	HR = 0·49, *p* = 0·007	7% vs. 0%	2007 (FDA)
	RCC	NCT00098657 NCT00083889 [33, 34]	Phase III, sunitinib vs. INF-a	HR = 0.42, *p* < 0.001	HR = 0.82, *p* = 0.05	31% vs. 6%	2007 (FDA)
	pNETs	NCT00428597 [35]	Phase III, sunitinib vs. placebo	HR = 0.42, *p* < 0.001	NA	9.3% vs. 0%	2011 (FDA)
Pazopanib (GlaxoSmith Kline)	RCC	NCT00720941 [37]	Phase III, pazopanib vs. placebo	HR = 0.46, *p* < 0.0001	NA	30% vs. 3%	2009 (FDA)
	STS	NCT00753688 [42]	Phase III, pazopanib vs. placebo	HR = 0.31, *p* < 0.0001	HR = 0.86, *p* = 0.25	9% vs. 0%	2012 (FDA)
Axitinib (Pfizer)	RCC	NCT00678392 [43, 44]	Phase III, axitinib vs. sorafenib	HR = 0.67, *p* < 0.0001	HR = 0.97, *p* = 0.37	19% vs. 9%	2012 (FDA)
Regorafenib (Bayer)	CRC	NCT01103323 [47]	Phase III, regorafenib vs. placebo	HR = 0.49, *p* < 0.0001	HR = 0.77. *p* = 0.005	1.0% vs. 0.4%	2012 (FDA)
		NCT01584830 [48]	Phase III, regorafenib vs. placebo	HR = 0.31, p < 0.0001	HR = 0.55, *p* = 0.0002	4% vs. 0%	
	GIST	NCT01271712 [49]	Phase III, regorafenib vs. placebo	HR = 0.27, *p* < 0.0001	HR = 0.77, *p* = 0.199	4.5% vs. 1.5%	2013 (FDA)
	HCC	NCT01774344 [50]	Phase III, regorafenib vs. placebo	HR = 0.46, *p* < 0.0001	HR = 0.63, *p* < 0.0001	11% vs. 4%	2017 (FDA)
Cabozantinib (Exelixis)	MTC	NCT00704730 [53]	Phase III, cabozantinib vs. placebo	HR = 0.28, *p* < 0.001	HR = 0.98	28% vs. 0%	2012 (FDA)
	RCC	NCT01865747 [55, 56]	Phase III, cabozantinib vs. everolimus	HR = 0.58, *p* < 0.001	HR = 0.66, *p* = 0.0003	21% vs.5%	2016 (FDA)
Nintedanib (Boehringer)	IPF	NCT00514683 [60]	Phase II, nintedanib vs. placebo	NA	NA	NA	2014 (FDA)
		NCT01335464 NCT01335477 [61]	Phase III, nintedanib vs. placebo				
	NSCLC	NCT00805194 [63]	Phase III, docetaxel + nintedanib vs. docetaxel + placebo	HR = 0.79, *p* = 0.0019	HR = 0.94, *p* = 0.27	4.4% vs. 3.3%	2014 (EMA)
Lenvatinib (Eisai )	DTC	NCT01321554 [67]	Phase III, lenvatinib vs. placebo	HR = 0.21, *p* < 0.001	HR = 0.73, *p* = 0.10	64.8% vs. 1.5%	2015 (FDA)
	RCC	NCT01136733 [68]	Phase II, lenvatinib + everolimus vs. lenvatinib vs. everolimus	HR = 0.4, *p* = 0.0005^*^	HR = 0.51, *p* = 0.024^*^	43% vs. 27% vs. 6%	2016 (FDA)
	HCC	NCT01761266 [69]	Phase III, lenvatinib vs. sorafenib	HR = 0.64, *p* < 0.0001	HR = 0.92	40.6% vs. 12.4%	2018 (FDA)
Apatinib (Hengrui)	GC	NCT01512745 [73]	Phase III, apatinib vs. placebo	HR = 0.709, *p* = 0.015	HR = 0.444, *p* < 0.001	2.8% vs. 0%	2014 (CFDA)
Anlotinib (Chia-taiTianqing)	NSCLC	NCT02388919 [76]	Phase III, anlotinib vs. placebo	HR = 0.25, *p* < 0.001	HR = 0.68, *p* = 0.002	9.2% vs. 0.7%	2018 (CFDA)
Fruquintinib (Hutchison)	CRC	NCT02314819 [80]	Phase III, fruquintinib vs. placebo	HR = 0.26, *p* < 0.001	HR = 0.65, *p* < 0.001	4.7% vs. 0%	2018 (CFDA)

*Abbreviation*: *RCC* renal cell carcinoma, *HCC* hepatocellular carcinoma, *DTC* differentiated thyroid cancer, *GIST* gastro-intestinal stromal tumor, *pNETs* pancreatic neuroendocrine tumors, *STS* soft tissue sarcoma, *CRC* colorectal cancer, *MTC* medullary thyroid cancer, *IPF* idiopathic pulmonary fibrosis, *NSCLC* non-small cell lung cancer, *GC* gastric cancer, *PFS* progression-free survival, *OS* overall survival, *ORR* objective response rate, *NA* not available, *FDA* US Food and Drug Administration, *CFDA* China Food and Drug Administration, *EMA* European Medicines Agency

*Lenvatinib + everolimus vs. everolimus

### Sorafenib

Sorafenib is the first anti-angiogenic receptor TKI, targeting VEGFR-1/2/3, PDGFR-β, and c-Kit receptor. It was initially approved for the treatment of advanced renal cell carcinoma (RCC) based on a phase III, randomized, double-blind clinical trial [27]. As many as 903 patients who are resistant to standard therapy were randomly assigned into two groups: sorafenib or placebo. The study demonstrated a significant improvement in median progression-free survival (PFS) in sorafenib group compared with placebo group (5.5 vs. 2.8 months, *p* < 0.001), and the partial response was elevated from 2% to 10% (*p* < 0.001) [27]. The medium overall survival (OS) demonstrated a reduced risk of death among patients receiving sorafenib though a statistics discrepancy did not reach. The approval of sorafenib by the FDA in 2007 in advanced hepatocellular carcinoma (HCC) was based on the result of SHARP trial [28]. It demonstrated that both the median OS and time to radiologic progression were nearly 3 months longer in sorafenib group than that in placebo group. Now, sorafenib is recognized as a standard treatment for patients with advanced HCC. Sorafenib also showed antitumor activity in differentiated thyroid cancer (DTC). The FDA approved sorafenib in radioactive iodine (RAI) refractory DTC in November 2013 based on the encouraging results of DECISION trial [29], and it was the first target therapy for this type of cancer. A total of 417 patients were enrolled and randomly assigned to sorafenib group or placebo group. PFS was significantly improved in sorafenib arm compared with placebo arm while the OS showed no significant difference in these two groups. Adverse events (AEs) related to sorafenib in these three kinds of carcinomas were similar, mainly including diarrhea, fatigue, desquamation, and hand-foot skin reaction [27–29]. Sorafenib in combine with gemcitabine acquired a favorable result for advanced pancreatic cancer in a phase I trial but failed to demonstrate positive result in phase III trial [30].

### Sunitinib

Sunitinib, the second approved anti-angiogenic receptor TKI, binds to VEGFR-1/2/3, PDGFR-α/β, c-Kit receptor, Fms-like tyrosine kinase-3 receptor (FLT-3), and receptor encoded by the ret proto-oncogene (Ret) [31]. It was the first cancer drug simultaneously approved by the FDA for two different indications: imatinib-resistant gastrointestinal stromal tumor (GIST) and RCC. In the pivotal phase III study, advanced GIST patients who failed imatinib therapy were treated in a randomized and blinded fashion with either sunitinib or placebo [32]. The result revealed a prolongation of time to progression from 6.4 weeks to 27.3 weeks (*p* < 0.0001), and the objective response rate (ORR), although relatively low, was significantly higher in the sunitinib than that in the placebo group (7% vs. 0%, *p* = 0.006) [32]. Additionally, OS obtained from initial sunitinib treatment was better than the placebo group. The landmark trial of sunitinib as a standard of care for first-line advanced RCC was the phase III study of sunitinib versus interferon alfa-2a reported in 2007, in which the superiority of sunitinib in terms of response rate, PFS, and OS were reported [33, 34]. The most common side effects related to sunitinib were diarrhea, fatigue, nausea, and skin discoloration in these two kinds of carcinoma [32, 34]. Beyond that, in May 2011, the FDA approved sunitinib for treating patients with advanced progressive pancreatic neuroendocrine tumors (pNETs) based on the results of a phase III study [35]. The study was terminated early on account of the notable better outcome in sunitinib group with the consent of the Independent Data Committee. The PFS was longer in the sunitinib group than that of the placebo group (11.4 vs. 5.5 months, *p* < 0.001), and the ORR was higher in the sunitinib group (9.3% vs. 0%, *p* < 0.007). Though the median OS was not reach at the cutoff point, the HR is in favor of sunitinib [35].

### Pazopanib

Pazopanib is a multi-kinase inhibitor on VEGFR-1/2/3, PDGFR-α/β, and c-Kit receptor [36]. The study VEG105192 conducted by Sternberg et al. compared pazopanib to placebo in 435 advanced RCC patients [37]. The results indicated pazopanib significantly improved the PFS and ORR rate compared with placebo; thereby, pazopanib was approved by the FDA in 2009 for advanced RCC patients. As both pazopanib and sunitinib have shown benefit in patients with RCC as first-line treatment, the efficacy and safely comparison between them were worth of consideration. The COMPARZ study was the first head-to-head phase III trial comparing sunitinib vs. pazopanib as first-line treatment for advanced RCC patients [38]. This study showed PFS was not inferior in the pazopanib group compared to the sunitinib group, and the ORR was higher in the pazopanib group (*p* < 0.05) and the OS between these two groups showed no statistical significance. Guo et al. further analyzed the safety of pazopanib and sunitinib in Asian and non-Asian subpopulations, indicating well-tolerated in both subpopulations [39]. In addition to the COMPARZ trial, the PISCES study adopted patients’ preference as the primary end point, and the result demonstrated patients favored pazopanib over sunitinib on account of the toxicity profile and health-related quality of life (HRQoL) score [40]. Noticeably, tolerability profiles were different between two drugs despite they shared the similar target pathways that is pazopanib has a lower incidence of 3/4 grade fatigue, hand-foot syndrome, and myelosuppression but more frequent hepatic injury [41]. Such discrepancy in toxicities should be considered for patients with specific conditions. Additionally, based on the results of PALETTE trial, pazopanib was approved by the FDA as a treatment for advanced soft tissue sarcoma (STS) in April 2012 [42]. As many as 369 metastatic non-adipocytic STS patients who failed after the standard chemotherapy were randomly assigned to receive pazopanib or placebo in a 2:1 ratio. Both the median PFS and OS were longer in the pazopanib group compared with placebo arm (PFS 4.6 vs. 1.6 months, *p* < 0.0001; OS 12.5 vs. 10.7 months, *p* = 0.2514). The response rate was 0% in the placebo group and 9% in the pazopanib group [42].

### Axitinib

Axitinib, targeting VEGFR-1/2/3, PDGFR-α/β, and c-Kit receptor, was approved by the FDA in 2012 for the second-line treatment of patients with advanced RCC [41]. The phase III AXIS study indicated that the median PFS was longer in the axitinib group compared to the sorafenib group (6.7 vs. 4.7 months, *p* < 0.0001) and the ORR was also higher in the axitinib arm [43]. According to the update results of the AXIS trial, no difference of OS was found between these two arms [44]. The most commonly treatment-related side effects were diarrhea, hypertension, and fatigue, and the incidence of hypertension appears higher in axitinib than other TKIs [45].

### Regorafenib

Regorafenib could inhibit a number of key angiogenic RTKs, including VEGFR-1/2/3, PDGFR-α/β, FGFR-1/2, Tie2, and c-Kit receptor [46]. It had demonstrated clinical effectiveness in patients with metastatic colorectal cancer (mCRC) who had progress after prior standard therapy and approved by the FDA in 2012. Two large randomized phase III trials, CORRECT and CONCUR, demonstrated that regorafenib prolonged the median OS and PFS in mCRC patients, and the drug-related adverse events were manageable [47, 48]. Based on the results of phase III GRID clinical trial [49], the FDA expanded the indication of regorafenib to patients with advanced GIST following the failure of imatinib and sunitinib in 2013. In this study, regorafenib improved the PFS to 4.8 months and the placebo arm was just 0.9 months, but no difference in the OS were observed between these two groups (HR = 0.77, *p* = 0.199). The most common regorafenib-related AEs of grade 3 or higher were hypertension (23%), hand-foot skin reaction (20%), and diarrhea (5%) [49]. Recently, a large phase III clinical trial (RESORCE) laid the foundation of the approved of regorafenib in HCC [50]. In RESORCE trial, 573 patients who failed or tolerated sorafenib were enrolled, and the results demonstrated that regorafenib significantly prolonged OS (10·6 vs. 7.8 months, *p* < 0.0001) and PFS (3.1 vs. 1.5 months, *p* < 0.0001). The most common AEs were hand-foot skin reaction, diarrhea, and fatigue. Based on the result of RESORCE trial, the FDA approved the regorafenib for the second line HCC [51].

### Cabozantinib

Cabozantinib is a small pan-tyrosine kinase inhibitor for VEGFR-1/2/3, c-Kit receptor, c-Met, and FLT-3 [52]. The approval of cabozantinib by the FDA in November 2012 for metastatic medullary thyroid cancer (MTC) was based on a phase III trial (EXAM) [53]. In this study, 330 patients with progressive metastatic MTC were randomly assigned to cabozantinib arm or placebo arm. The study reached its primary end point by indicating an improvement of PFS in cabozantinib arm (11.2 vs. 4.0 months, *p* < 0.001). The ORR was 28% with cabozantinib compared to 0% with placebo, while no statistically significant difference in OS was observed between these two arms. The most common AEs were diarrhea and palmar-plantar erthyrodysesthesia syndrome [54]. Cabozantinib also demonstrated its antitumor efficacy compared with everolimus in mRCC who had progressed after VEGFR-targeted therapy. The pivotal phase III METEOR trial had leaded the approval by the FDA for advanced mRCC as second-line treatment [55, 56]. According to the result, the median PFS was 7.4 months in the cabozantinib group and 3.8 months in the everolimus group, and ORR was increased by 16% in cabozantinib group (21% vs. 5%, *p* < 0.001) [55]. More importantly, the final updated results show a significant improvement in OS with cabozantinib (21.4 months vs. 16.5 months, *p* = 0.0003) [56]. More importantly, cabozantinib was expected to become a novel systemic therapy for patients with metastatic hepatocellular carcinoma (mHCC) based on the positive result of CELESTIAL trial [57]. The study showed that treatment with cabozantinib resulted in longer OS (10.2 vs. 8.0 months, *p* = 0.005) and PFS (5.2 vs. 1.9 months, *p* < 0.001), and the ORR was also higher in cabozantinib arm (4% vs. 1%, *p* = 0.009) [57]. Encouragingly, cabozantinib garnered FDA approval for mHCC patients after the failure of sorafenib in January 2019.

### Nintedanib

Nintedanib is a multiple angio-kinase inhibitor targeting VEGFR-1/2/3, PDGFR-α/β, and FGFR-1/2 [58]. It has initially approved by the FDA for the treatment of idiopathic pulmonary fibrosis (IPF) [59] depending on the TOMORROW trial [60] and INPULSIS-1/2 trial [61]. In the same year, nintedanib combined with docetaxel therapy was approved for non-squamous non-small cell lung cancer (NSCLC) patients as a second-line treatment by the European Medicines Agency (EMA) but not by the FDA [62]. In a pivotal randomized, double-blind phase III trial (LUME-Lung 1), PFS was improved in the docetaxel plus nintedanib group in relative to placebo plus docetaxel (3.4 vs. 2.7 months, *p* = 0.0019) [63], while the OS showed no discrepancy (10.1 months vs. 9.1 months, *p* = 0.2720). Interestingly, analysis in patients with adenocarcinoma histology showed a prolonged PFS (HR = 0.77, *p* = 0.0193) and OS (HR = 0.83, *p* = 0.0359) in the docetaxel plus nintedanib group. AEs were more common in the docetaxel plus nintedanib group compared with the docetaxel plus placebo group with diarrhea, decreased neutrophils, and fatigue. Subsequently, Hanna et al. conducted a phase III trial, LUME-Lung 2 study, comparing the efficacy of nintedanib plus pemetrexed in patients with NSCLC exclusively [64]. Although the clinical trial was stopped prematurely, a PFS advantage was observed in the nintedanib plus pemetrexed group in the subsequent analysis. However, the subgroup analysis in adenocarcinoma histology showed no discrepancy between two arms, which was inconsistent with the findings in LUME-Lung 1 study. The real value of nintedanib in the second-line therapy of advanced NSCLC remained unclear.

### Lenvatinib

Lenvatinib is a kinase inhibitor targeting VEGFR-1/2/3, PDGFR-α/β, FGFR-1/2/3, Ret, and c-Kit [65]. It was first approved for patients with advanced RAI refractory DTC by the FDA based on the results of the SELECT study [66, 67]. In this study, lenvatinib significantly prolonged the PFS (18.3 vs. 3.6 months, *p* < 0.001) and improved the ORR (64.8% vs. 1.5%, *p* = 0.001). The most frequent side effects were hypertension, diarrhea, and fatigue/asthenia. Considering the dramatic efficacy and manageable side effects, the FDA expedited the approval of lenvatinib for RAI-refractory DTCs [65]. Encouragingly, it also showed anti-neoplastic activity in other solid tumors, including advanced RCC and HCC. Lenvatinib gained the FDA approval for the treatment of metastatic RCC based on the positive result of a phase II clinical trial [68]. The study was designed to assess whether the combination of lenvatinib plus everolimus was superior compared to the single agents. The primary end point of PFS in the combination group was significantly longer than everolimus alone (14.6 vs. 5.5 months, *p* = 0.0005). The ORR in lenvatinib plus everolimus arm, lenvatinib alone arm, and everolimus alone arm were 43%, 27%, and 6%, respectively. Additionally, the incidence of grade 3–4 AEs was 71% in patients receiving lenvatinib plus everolimus, 79% in single-agent lenvatinib, and 50% in single-agent everolimus [68]. Based on these results, lenvatinib in combination with everolimus was recommended as a second-line systemic therapy in mRCC. Lenvatinib garnered the FDA approval for HCC in 2018 based on a series of clinical trials, among which a global phase III trial (REFLECT) was the most important [69]. This study aimed to assess the efficacy of lenvatinib vs. sorafenib as a first-line treatment for patients with unresectable HCC. The result was notable that the OS in lenvatinib group was non-inferior to the sorafenib group (13.6 vs. 12.3 months), and the PFS of lenvatinib group was longer than that of sorafenib (7.4 vs. 3.7 months, *p* < 0.0001) [69]. Lenvatinib also showed a greater ORR compared with the sorafenib group (24.1% vs. 9.2%, *p* < 0.0001), and the treatment-emergent AEs were tolerable in both groups [69, 70]. Lenvatinib, as the second first-line agent for advanced HCC patients, was a great breakthrough in the field of HCC in the past 10 years.

### Apatinib

The past decade has also witnessed the great progress in the development of anti-tumor drugs developed by Chinese researchers. Apatinib can simultaneously suppress the kinase activities of VEGFR-2, c-Kit, and c-Src and is approved by the CFDA for the treatment of advanced gastric cancer (GC) in October 2014 [71, 72]. The efficacy and safety profile of apatinib in patients with metastatic gastric or gastroesophageal junction adenocarcinoma who had failed at least two lines of chemotherapy was evaluated in a series of clinical trials. A phase III randomized clinical trial, conducted by Li and collaborators, has indicated its important role in three or more lines for GC patients [73]. The primary end points of OS and PFS were significantly prolonged by apatinib (OS 6.5 vs. 4.7 months; PFS 2.6 vs. 1.8 months). Though the ORR showed no difference between two groups, the disease control rate (DCR) favored apatinib over placebo treatment. The major treatment-related grade 3–4 AEs in apatinib arm included hand-foot syndrome, proteinuria, and hypertension [73].

### Anlotinib

Anlotinib targets VEGFR-1/2/3, FGFR-1–4, PDGFR-α/β, c-Kit, and Ret [74]. The ALTER0302 trial demonstrated 3.6 months longer PFS in NSCLC patients receiving anlotinib [75]. Soon after, the ALTER0303 trial was conducted, further confirming the drug’s efficacy in advanced NSCLC [76]. The results showed that both OS (9.6 vs. 6.3 months, *p* = 0.002) and PFS (5.4 vs. 1.4 months, *p* < 0.001) were significantly longer in the anlotinib group compared with placebo. Anlotinib also produced significant ORR and DCR benefits vs. placebo and had a manageable safety profile [76, 77]. It was approved by the CFDA as a third-line or further therapy for advanced NSCLC patients in 2018. Until now, apatinib and anlotinib have not gained the approval of the FDA, but both of them were identified as orphan drugs in the USA.

### Fruquintinib

Fruquintinib is a potent small molecule inhibitor of VEGFR-1/2/3 [78]. In the phase II clinical trials, fruquintinib showed a significant PFS benefit in patients with treatment-refractory mCRC [79]. Then, a randomized, double bind, phase III (FRESCO) trial conducted by Li et al. laid the foundation for the approval of this drug on patients with mCRC by the CFDA in 2018 [80]. In this study, mCRC patients who had progressed after at least two lines of chemotherapy were allocated to receive either fruquintinib or placebo. The primary end point median of OS was significantly longer in the fruquintinib group compared to placebo (9.3 vs. 6.6 months, *p* < 0.001), and the median PFS also significantly increased by fruquintinib (3.7 vs. 1.8 months, *p* < 0.001). Moreover, higher ORR and DCR were observed in patients receiving fruquintinib with a manageable safety profile. Additionally, a phase I clinical trial is ongoing in the USA, exploring the efficacy and safety in non-Chinese populations [81].

## Novel anti-angiogenic TKIs under investigation

While the approved anti-angiogenic TKIs are trying to expand their indication in other cancer types, numerous new anti-angiogenic TKIs are also being extensively explored. Three representative TKI drugs with potential to be approved in the near future are presented.

Motesanib, also named AMG 706, is an orally multi-targeted inhibitor of VEGFR-1/2/3, PDGFR-α/β, and c-Kit [82]. Motesanib was considered as a potent anti-tumor drug in Asian advanced NSCLC patients based on the subgroup analysis of MONET1 trial [83]. However, the results of later phase III trial (MONETA) were disappointing with no advantage in patients receiving motesanib plus paclitaxel and carboplatin over placebo plus paclitaxel and carboplatin [84]. Nevertheless, two phase II trials have indicated remarkable anticancer activity of motesanib among patients with advanced thyroid cancer [85, 86]. Recently, Lubner et al. examined the efficacy of motesanib in low-grade NETs in a phase II trial [87]. The study reached its primary objective with a 4-month PFS of 78.5%, and the median PFS in all patients was 8.7 months. All in all, motesanib is as potential as a systemic targeted therapy for NETs, but its niche in the treatment of NETs still needs further study.

Cediranib, also named AZD-2171, is a potent inhibitor of VEGF signaling that binds all three VEGFR (VEGFR-1/2/3), together with c-Kit and PDGFR-α/β [88]. Though, cediranib had failed phase III clinical trials in NSCLC [89], mCRC [90, 91], and recurrent glioblastoma [92], it showed new hope in recurrent ovarian cancer. The ICON6 trial evaluated the efficacy and safety of cediranib plus platinum-based chemotherapy and as continued maintenance treatment in patients with relapsed platinum-sensitive ovarian cancer [93]. Unfortunately, the ICON6 trail was prematurely terminated on account of the depressing results in other cancer types. Finally, a total of 486 women were randomly allocated to arm A (chemotherapy plus placebo 6 cycles and continued placebo), arm B (chemotherapy plus cediranib 6 cycles and switched to placebo), and arm C (chemotherapy plus cediranib 6 cycles and continued cediranib). The median PFS was significantly longer in arm C at 11 months, compared to 8.7 months in arm A (*p* < 0.0001) [93], and the OS was 7.4 months higher in arm C compared to arm A (*p* = 0.21) [94]. Most common side effects of grade 3–4 in arm C were neutropenia, fatigue, and hypertension during the chemotherapy phase and diarrhea, fatigue, and neutropenia during maintenance treatment.

Sulfatinib is a multi-target TKI targeting VEGFR-1/2/3, FGFR1, and colony stimulating factor 1 receptor (CSF1R). A phase I study (NCT02133157) observed an acceptable safety profile and encouraging antitumor activity in patients with advanced solid tumors, particularly in NETs [95]. At present, one phase II study (NCT02267967) and two phase III studies (NCT02589821, NCT02588170) conducted on advanced NETs are ongoing [96].

## Anti-angiogenic receptor TKIs in combination with immunotherapy

Immunotherapy has been changing the paradigm of oncology treatment in the recent years [97–99]. Whether the combination of TKIs and immunotherapy can create synthetic effect is a hot topic. The emerging evidences suggest that anti-angiogenic therapy may not only inhibit neo-vascular formation, but also regulate the immune microenvironment [100]. This provided a theoretic basis for the combination of TKIs and immunotherapy. Subsequently, hundreds of clinical trials were designed to access the efficacy of combining TKIs with immune checkpoint blockade. A phase Ib study (JAVELIN Renal 100) conducted by Choueiri et al. interrogated the combination therapy of axitinib plus avelumab (a PD-L1 mAb) in advanced RCC patients [101]. The DCR reached 78% in 55 patients with three complete responses and the safety profile was manageable. These encouraging results supported the further study of these drug combinations. Now, the phase III JAVELIN Renal 101 trial finished [102]. The result showed that in 866 patients with mRCC, the axitinib plus avelumab group showed a remarkable improvement in median PFS compared with sunitinib (13.8 vs. 8.4 months, *p* < 0.0001), and the ORR were 51.4% and 25.7%, respectively. Furthermore, among patients with PD-L1+ tumors, the ORR and the PFS also favored the combination group [102]. The combination of axitinib and avelumab would be a promising strategy for patients with mHCC based on the positive result of JAVELIN Renal 101. Other combinations such as lenvatinib plus pembrolizumab or SHR 1210 plus apatinib in patients with HCC were also ongoing [103]. The combination of immunotherapy with TKIs has demonstrated promising outcome in a certain type of carcinomas, but further optimized combinations are required and caution must be taken to avoid severe toxicity.

## Future perspectives

The development of anti-angiogenic agents has attracted great attention. Bevacizumab, the first clinically approved anti-VEGF targeted agents, provides a first proof of principle of anti-angiogenic treatment in cancer. Though monotherapy with bevacizumab is largely inefficient, it really exerts therapeutic efficacy in various types of carcinoma when in combination with chemotherapy [104]. Because tumor angiogenesis is regulated by multiple pathways, many interconnected pathways can compensate the effect of single inhibition of VEGF signaling. It seems that multi-targeted TKIs hold a therapeutic advantage over monoclonal antibody as they can block multiple angiogenic signaling pathways simultaneously. Indeed, TKIs have shown their efficacy in many types of cancers, mainly RCC and HCC. Although all anti-angiogenic receptor TKIs share the same mechanism of action and the similar spectrum of targeted kinases, they are different in their pharmacokinetics and substance-specific AEs. The one possible explanation may be that the subtle difference on chemical structure leads to the variable affinity and potency to targets. Another possibility is that those TKIs may act on some unidentified targets beyond known kinases. With more and more anti-cancer agents available, it is a challenge for the oncologist to make an optimal choice in the sequence of treatment. For instance, 12 drugs have been approved for patients with HCC, including 6 anti-angiogenic TKIs until 2017 [105]. Though, the international guidelines have reached a global consensus for the choice of drugs in different lines. The optimal strategy and the sequence of drugs as well as the right time of the incorporation of other therapeutic methods such as surgery, radiology has not yet been resolved. Tolerance of receptor TKIs should also be taken into account.

Another challenge for anti-angiogenesis TKIs is the lack of robust biomarkers to identify patients with cancer who will benefit from anti-angiogenic therapy. Unlike RTK inhibitor, larotrectinib is special for cancer with tropomyosin receptor kinases (TRK) fusion-positive and has demonstrated significant efficiency in patients with different tumor histology [106]. One of the main problems in identifying such a biomarker for anti-angiogenic therapy may come from the complex feedback loops and cross talk between signaling pathways. Currently, some biomarkers have been proposed, such as VEGF, VEGFR-2, FGF-2, or IL-8, but none of them have yet been validated for routine clinical use [104]. Recently, a cohort study conducted by Liu et al. indicated a positive correlation between the anti-angiogenesis-related AEs and prolonged OS [107]. It means that side effects, such as high blood pressure, hypothyroidism, or hand-foot syndrome, may associate with the anti-tumor efficacy. Similarly, Rini et al. demonstrated that patients with diastolic blood pressure ≥ 90 mmHg had a longer OS and PFS [108]. As there are no molecular biomarkers available for clinical use, those side effects might be helpful for clinical decision.

The future of TKIs could be their positioning besides metastatic setting, such as in adjuvant therapy and neoadjuvant treatment. There were three well-known phase III clinical trials that explored the use of TKIs in RCC in adjuvant setting, namely ASSURE (adjuvant sunitinib vs. sorafenib vs. placebo), PROTECT (pazopanib vs. placebo), and S-TRAC (sunitinib vs. placebo) [109–111]. Only S-TRAC study showed a significant improvement by sunitinib in disease-free survival in high-risk RCC after nephrectomy [111]. Based on the result of S-TRAC trial, sunitinib was approved by the FDA as an adjuvant therapy for RCC patents in 2017. Unfortunately, adjuvant sorafenib for HCC patients reached a negative result [112]. The utilization of TKIs before surgery has also been studied. A phase II trial explored the safety and efficacy of the use of pazopanib prior to cytoreductive nephrectomy RCC patients, suggesting the safety, and clinical benefit could be expected [113]. The precision role of anti-angiogenic TKI in adjuvant and neoadjuvant therapy needs further investigation.

It is noted that the indication of these receptor TKIs are mainly restricted to highly vascular tumor, like RCC, HCC, NSCLC, and CRC. Their efficacy in other types of cancers needs further exploration [30]. In most case, anti-angiogenesis treatment increases the PFS of patients, while the increase in OS is unsatisfactory. Great breakthrough in immunotherapy brings new possibility for the combination of TKIs, and positive results in a certain type of carcinoma attract broad attention [114].

## Abbreviations

AEs: Adverse events
ANGPT: Angiopoietin
ATP: Adenosine triphosphate
CFDA: China Food and Drug Administration
DCR: Disease control rate
DTC: Differentiated thyroid cancer
EGF: Epidermal growth factor
FDA: US Food and Drug Administration
FGF: Fibroblast growth factor
GC: Gastric cancer
GIST: Gastro-intestinal stromal tumor
mCRC: Metastatic colorectal cancer
MTC: Metastatic medullary thyroid cancer
NETs: Neuroendocrine tumors
NSCLC: Non-small cell lung cancer
ORR: Objective response rate
OS: Overall survival
PDGF: Platelet-derived growth factor
PFS: Progression-free survival
PIGF: Placenta growth factor
PKIs: Protein kinase inhibitors
RAI: Radioactive iodine
RCC: Renal cell carcinoma
RTKs: Receptor tyrosine kinases
TKIs: Tyrosine kinase inhibitors
VEGF: Vascular endothelial growth factor
